# Reliability Analysis and Optimization of a Reconfigurable Matching Network for Communication and Sensing Antennas in Dynamic Environments through Gaussian Process Regression

**DOI:** 10.3390/s24092689

**Published:** 2024-04-24

**Authors:** Seppe Van Brandt, Kamil Yavuz Kapusuz, Joryan Sennesael, Sam Lemey, Patrick Van Torre, Jo Verhaevert, Tanja Van Hecke, Hendrik Rogier

**Affiliations:** Internet Technology and Data Science Lab, Department of Information Technology, Faculty of Engineering and Architecture, Ghent University and Imec, 9052 Gent, Belgiumjo.verhaevert@ugent.be (J.V.); hendrik.rogier@ugent.be (H.R.)

**Keywords:** Internet of Things (IoT), matching network, algorithm, reliability, Gaussian process, regression analysis, statistical analysis

## Abstract

During the implementation of the Internet of Things (IoT), the performance of communication and sensing antennas that are embedded in smart surfaces or smart devices can be affected by objects in their reactive near field due to detuning and antenna mismatch. Matching networks have been proposed to re-establish impedance matching when antennas become detuned due to environmental factors. In this work, the change in the reflection coefficient at the antenna, due to the presence of objects, is first characterized as a function of the frequency and object distance by applying Gaussian process regression on experimental data. Based on this characterization, for random object positions, it is shown through simulation that a dynamic environment can lower the reliability of a matching network by up to 90%, depending on the type of object, the probability distribution of the object distance, and the required bandwidth. As an alternative to complex and power-consuming real-time adaptive matching, a new, resilient network tuning strategy is proposed that takes into account these random variations. This new approach increases the reliability of the system by 10% to 40% in these dynamic environment scenarios.

## 1. Introduction

The Internet of Things (IoT) constitutes a network of interconnected devices that seamlessly communicate and share data, fostering an intelligent and responsive environment [1,2]. IoT resulted from the convergence of advancements in communication technologies, embedded systems, and sensor networks. The integration of these elements has given rise to dynamic ecosystems where devices seamlessly interact with one another, in both domestic as industrial applications [3,4,5,6]. For the implementation of Internet of Things to be viable, the connectivity of interconnected devices needs to be robust and should not be compromised by environmental factors and deployment conditions. The integration of antennas in smart devices [7], or behind the surface of a wall or a desk [8,9], brings antennas required for communication and/or sensing closer to the user and other objects, possibly diminishing their radiation performance due to body parts or objects in the near field of such antennas [10].

Reconfigurable matching networks and filters have been proposed as an interface between the antenna and the circuit to ensure a reliable communication link and good sensing capability and to protect the RF amplifier when the antenna impedance is detuned due to environmental factors [11,12]. These implementations leverage various technologies and each has its own advantages and disadvantages. First, PIN diode switches [13,14] can only reconfigure to certain discrete states and suffer from diode losses. Additionally, MEMS switches [12,15] and MEMS varactors [16] use mechanical movement to tune the circuit, which can reduce the size and losses, but come at a greater cost and require higher control voltages. Another approach involves employing CMOS technology [11,17], providing compact and energy-efficient solutions but at a high production cost. An alternative strategy, utilizing varactor diodes [18,19,20,21,22,23], provides an effective trade-off between cost, losses, and tuning capability. In [24], researchers introduced an air-filled substrate-integrated waveguide (AFSIW) antenna that was co-designed with a tuneable matching network, based on three varactor diodes, for integration in smart surfaces.

Different (automatic) tuning algorithms have been proposed to steer matching networks in narrowband applications by performing tuning at certain predefined frequencies only [25,26,27,28,29]. This essentially makes these algorithms only applicable to narrowband scenarios. In [30], the proposed adaptive matching algorithm also performed tuning at only one frequency. This algorithm was ultimately applied to a broadband scenario. However, the frequency dependency of the load impedance, which poses an extra challenge in real-life applications, was not taken into account.

When confronted with dynamic changes in antenna impedance, a real-time response that counters these fluctuations as they occur may be implemented. For example, in [29], researchers were able to guarantee an antenna reflection coefficient below −14 dB for 87–95% of the time for a 406 MHz narrowband antenna worn by a rescue operator. Yet, such an approach consumes a lot of power and resources and, especially when trying to match to a frequency-dependent impedance over a broad frequency band, depending on how fast these changes happen, it could be hard for the adaptive algorithm that controls the matching network to follow these variations.

Instead of adopting a deterministic matching strategy that tries to follow these changes in real time, we propose a stochastic strategy that optimizes the antenna performance for a broad array of possible environmental configurations, thus making the system more resilient in a dynamic environment without extra power consumption and without the algorithm having to keep up with the changes in real time. Another benefit of spreading out the network matching routines in time concerns the corruption of the encoded message in the transmitted or received signal by unwanted amplitude or phase modulation during the matching process [31]. Additionally, the antenna impedance, as measured by the deployed system, can be unreliable. By optimizing over a range of impedances at once, these measurement errors become less significant.

In this research, the proposed algorithm is applied on a surface-embedded antenna that is detuned by the close presence of objects in order to compare the performance with a classic matching strategy. A multitude of different objects and configurations are chosen to represent realistic scenarios that a surface-embedded antenna could encounter.

In Section 2, the hardware is described, as well as the experimental setup and the measurement data. In Section 3, the Gaussian process regression method is briefly explained and applied to the data to characterize the reflection coefficient in a dynamic scenario. Additionally, a novel reliability optimization strategy is proposed in this section. The method performed to test the new optimization strategy, by means of simulation, is described in Section 4, and the results of these tests are shown and discussed in Section 5. Finally, in Section 6, this paper is ended with a conclusion.

## 2. Setup

### 2.1. Matching Network

We consider the scenario of a wireless communication or sensing system integrated in a piece of furniture, such as an office table. Given its deployment in a typical IoT scenario, the antenna may become detuned due to nearby objects; this is a problem that can be corrected by a controllable matching network in between the antenna and the amplifier, which connects to the transceiver system of the communication or sensing device.

**Figure 1:**
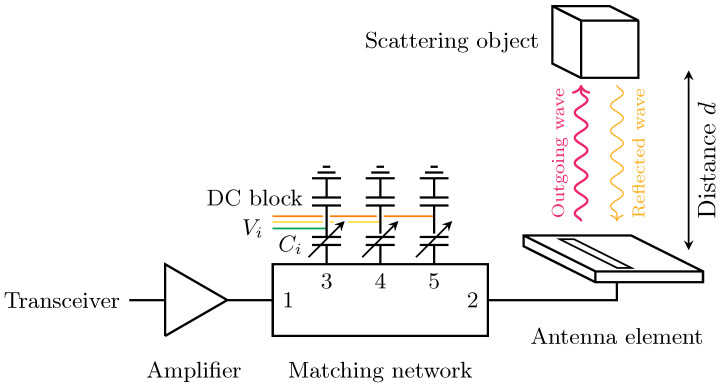
Schematic representation of the system setup. The amplifier is connected to the transceiver of the communication/sensing system. The matching network is deployed between the amplifier at port 1 and an antenna at port 2. It performs tuning via three varactors at ports 3, 4, and 5. Objects near the antenna reflect parts of the outgoing waves and detune the antenna, which is then compensated by controlling the capacitance of the varactors $C_{i}$, through voltages $V_{i}$.

**Figure 2:**
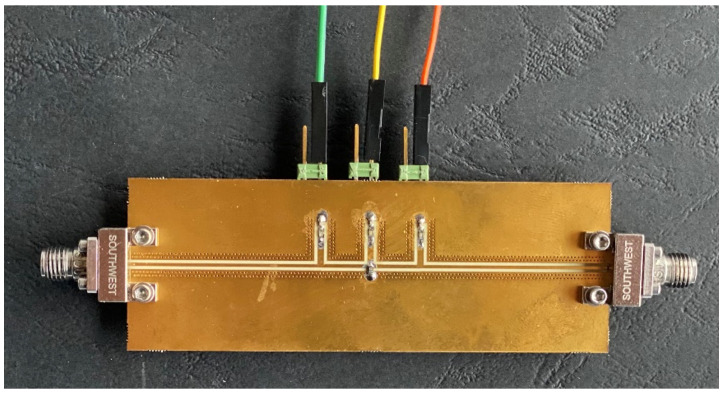
Photograph of the standalone matching network.

An antenna with the matching network is schematically shown in Figure 1. The adopted matching network, of which a photograph is shown in Figure 2, is identical to the one described in [24]. The system is implemented in grounded co-planar waveguide (GCPW) technology and is designed for IEEE 802.11 5 GHz bands between 5.15 GHz and 5.85 GHz, specifically the U-NII-1, U-NII-2, and U-NII-3 bands. Nevertheless, it is worth noting that various other applications also operate within this frequency range. The architecture of the matching network is based on a third-order Chebychev filter and consists of five ports: port 1 is connected to the amplifier, port 2 is connected to the antenna, and ports 3, 4, and 5 are terminated by varactors, whose capacitance is controlled by DC voltages, located on shorted λ/4 stubs and connected to the ground via a capacitor as a DC blocker. The third-order topology was adopted as a compromise between tuning range and losses.

The network can be described by a two-port S-matrix that is dependent on the impedance values of the varactors that are connected to ports 3, 4, and 5. The capacitances $C_{i}$ of the three varactors are controlled by the voltages $V_{i}$. They enable tuning by modifying the S-matrix of the two-port network under study.

### 2.2. Antenna Element

**Figure 3:**
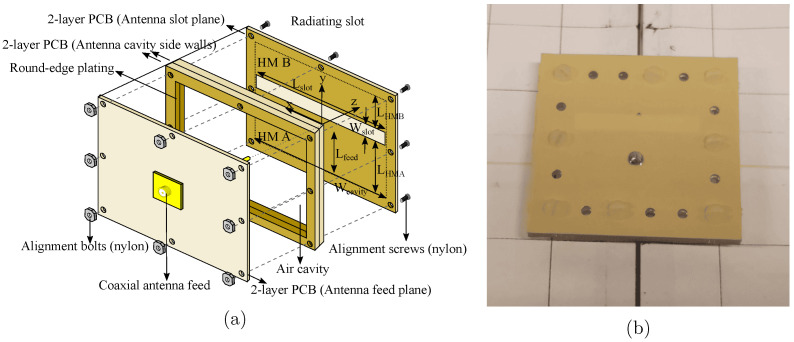
Exploded-view drawing (**a**) and a photograph of the standalone antenna element (**b**). The antenna element has a size of 62 mm by 60.5 mm. Antenna dimensions: L_slot_ = 38 mm, W_slot_ = 5.5 mm, W_cavity_ = 42 mm, L_HMA_ = 24 mm, L_HMB_ = 11 mm, L_feed_ = 11.85 mm.

The proposed antenna, depicted in Figure 3, is designed for operation in the 5.15 GHz to 5.85 GHz frequency band. It consists of two air-filled substrate-integrated waveguide (AFSIW) half-mode rectangular cavities (HM A and HM B), combined to form a cavity-backed slot antenna [32]. The distance between the half-mode cavities is determined by the width of the slot (W_slot_). This topology provides a large bandwidth, which allowed us to cover the entire 5.15 GHz to 5.85 GHz range [33]. Additionally, AFSIW technology allows for a large front-to-back-ratio and excellent environmental isolation [9].

The fabrication was realized by leveraging standard PCB manufacturing techniques on low-cost PCB substrates. The cavity was constructed by stacking two milled-out and round-edge plated 1.55 mm thick FR-4 substrates on top of each other, thus creating an air-cavity, as indicated in Figure 3a. Next, the antenna slot and feed plane, both implemented using a 0.254 mm thick ITERA laminate, are placed on the top and bottom sides of the air substrate. A 1.3 mm copper pin feeds the antenna via a coaxial connector and a slot in the top layer acts as the antenna’s aperture through which it radiates. Finally, nylon screws and bolts are positioned to maintain the alignment of all layers.

**Figure 4:**
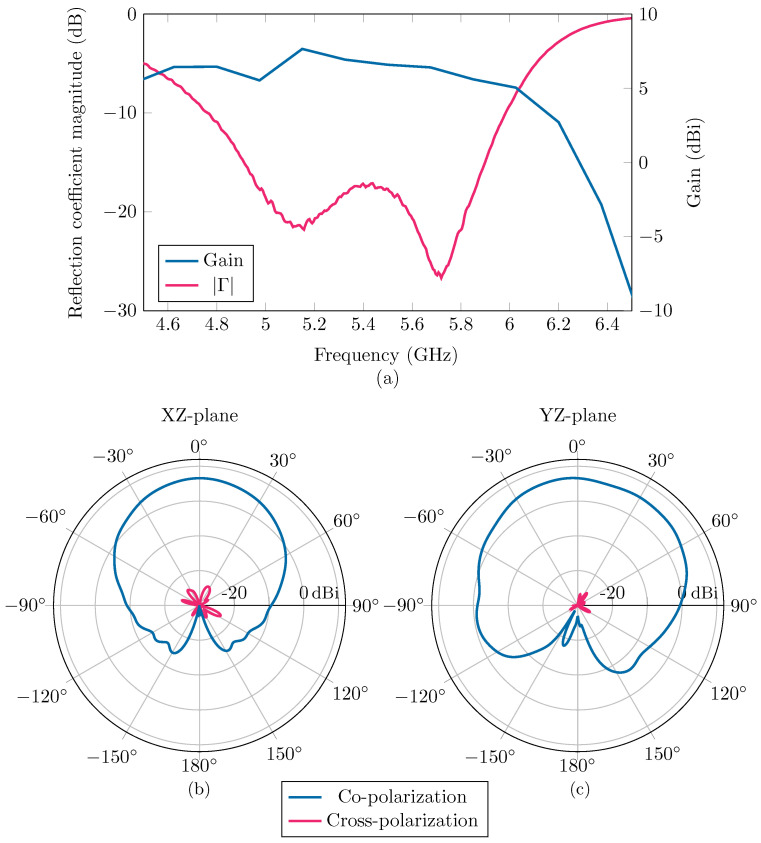
In (**a**), measurements of the reflection coefficient Γ and broadside realized gain of the standalone antenna are shown. The measured radiation pattern of the co- and cross-polarizations of the standalone antenna in the XZ-plane and the YZ-plane are shown in (**b**,**c**), respectively. These measurements were performed in free space, without the presence of scattering objects.

The antenna prototype was measured using a Keysight N5242B PNA-X Microwave Network Analyzer and an NSI MI near-field antenna system. Figure 4a demonstrates that the reflection coefficient magnitude |Γ| of the antenna radiating into free space stays below −17 dB within the 5.15 GHz to 5.85 GHz band. Additionally, within this band, the measured antenna gain consistently exceeds 5.5 dBi. The measured co- and cross-polarization radiation patterns in the XZ- and YZ-planes at the center frequency of 5.5 GHz are shown in Figure 4b,c. The antenna achieves a maximum gain of 6.58 dBi in the broadside direction. The half-power beamwidth (HPBW) in the XZ-plane equals 69° and 107° in the YZ-plane. Finally, the front-to-back ratio (FTBR) exceeds 30 dB while the cross-polarization level remains below −20 dB over the entire HPWB.

By connecting the antenna to port 2, the network further reduces to a one-port network. As a way to quantify the performance of the system, the reflection coefficient $S_{11}$ and transducer gain $G_{T}$ are used [34,35]. These parameters can be calculated for a one-port network with

(1) $$S_{11}=S_{11}^{\prime }+\frac{S_{12}^{\prime }S_{21}^{\prime }\Gamma }{1-S_{22}^{\prime }\Gamma }$$

and

(2) $$G_{T}={\left|S_{21}^{\prime }\right|}^{2}\frac{1-{\left|\Gamma \right|}^{2}}{|1-S_{22}^{\prime }{\Gamma |}^{2}},$$

where the $S_{ij}^{\prime }$ parameters correspond to the two-port network obtained by terminating ports 3, 4, and 5. When the reflection coefficient Γ at port 2 varies due to changes in the antenna environment, the $S_{ij}^{\prime }$ values must be modified as well to retain acceptable $S_{11}$ and $G_{T}$ values. This is achieved by tuning the varactor capacitances through their connected voltages.

### 2.3. Experimental Setup and Results

The experiment was designed to be representative of typical situations that surface-embedded antennas encounter in their daily usage. To characterize the effects of the presence of various objects within the near field of the antenna, experiments were conducted by placing objects directly above the antenna at six different vertical distances, $d\in \left[0\;\mathrm{mm},150\;\mathrm{mm}\right]$, and measuring the reflection coefficient Γ at the antenna port over the frequency band $f\in \left[5.15\;\mathrm{GHz},5.85\;\mathrm{GHz}\right]$. The measurements were restricted to the operational band of the matching network and antenna, since matching the system outside of this frequency range would not be achievable anyhow.

**Figure 5:**
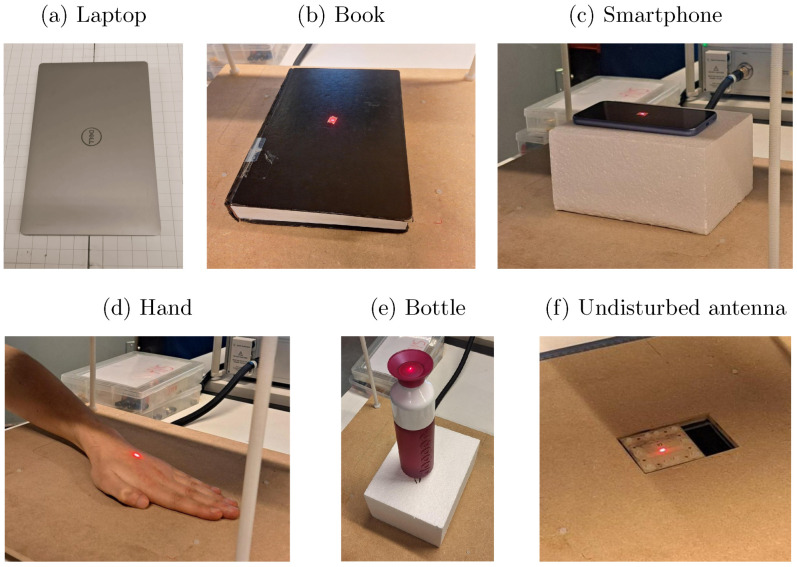
In (**a**–**e**), photos of the different objects during the measurements are shown. The smartphone is only shown in its orientation parallel to the antenna slot and only the completely filled bottle is shown. A laser pointer, positioned directly above the antenna slot, was used to accurately position the objects in the XY-plane, except in the measurements with the laptop where a grid surface was used for this purpose. In (**c**,**e**), the application of styrofoam is shown as it functions as a spacer to alter the height of the objects above the antenna. In (**f**), the positioning of the antenna within the measurement setup can be seen. The dimensions of the objects are as follows: laptop (376 × 251 mm), book (250 × 170 × 35 mm), smartphone (158 × 77 × 8 mm), hand (180 × 95 × 30 mm), and bottle (60 × 60 × 235 mm).

A careful selection of different objects were considered in this experiment. Specifically, the proximity effects of a laptop, a book, a smartphone, a hand, and a plastic water bottle on the antenna performance were evaluated experimentally. The laptop and smartphone were turned off during the measurements. Photographs of these objects in the measurement setup are shown in Figure 5. These objects were chosen because they are very common in situations where surface-integrated antennas could be used, such as the surface of a desk. Additionally, these objects have different electromagnetic properties. This way, the effects of various electromagnetic object characteristics can be studied. During the experiments, the objects were centered directly above the slot of the antenna. A vertical laser pointer or a paper grid was used to align the objects in the XY-plane and to ensure reproducibility. To better understand the influence of object orientation within this setting, two measurements were performed with the smartphone, one where the phone was aligned along the slot of the antenna (parallel to the *X*-axis), and one where the phone was perpendicular to the slot (parallel to the *Y*-axis). To study the influence of the presence of water in the bottle, the experiments were repeated with an empty, quarter-filled, half-filled, and full bottle. During the experiments, styrofoam blocks were placed between the antenna and the objects used to lift the objects above the antenna. Different thicknesses were used to achieve the different heights at which the measurements were performed. Styrofoam consists mostly of air and exhibits a relative permittivity close to one and a very low loss tangent. It has, therefore, a negligible effect on the radiating performance of the antenna. When conducting the measurements at a height equal to zero, no styrofoam was used and the objects were placed directly on top of the antenna.

**Figure 6:**
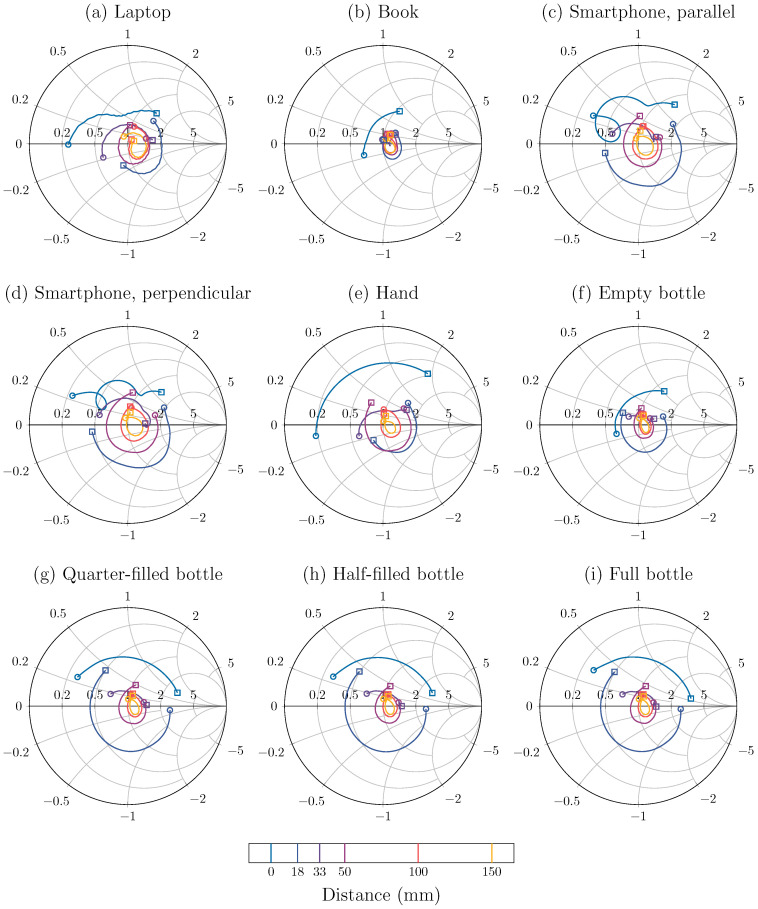
The measured reflection coefficient curves plotted on Smith charts, over the entire band at certain measured distances. The start of the band at 5.15 GHz is indicated by a circle, the end at 5.85 GHz by a square. To improve readability, the ripple on the measured curves was reduced with a Savitzky–Golay filter of order 2 with a window size of 20 MHz [36].

The reflection coefficient data stemming from this experiment, are shown in Figure 6. From these data, a clear dependency of the reflection coefficient on both the frequency and distance of the object is visible. At short distances, the influence of the object is the most significant, causing the biggest mismatch, which results in the largest magnitude of the reflection coefficient. As the distance becomes larger, the magnitude reduces rapidly and at distances beyond 50 mm, the magnitude does not change as much anymore and converges to the intrinsic, non-zero, reflection coefficient of the antenna. The reflection coefficient varies significantly with frequency and this will have to be taken into account when matching over the entire band is desirable.

## 3. Method

### 3.1. Gaussian Process Regression

In order to design and analyze a new matching strategy, it is essential that the variability in Γ in dynamic scenarios can be modeled in a realistic way. By using a compact model that reliably predicts Γ over the frequency band at various distances, this can be achieved. For this purpose, two Gaussian process regressions (GPRs) [37] were used. GPR is a supervised, probabilistic, non-parametric framework for regression analysis in which the constraint of having to chose a functional form of the regression is avoided. In this subsection, we focus on the laptop and the half-filled bottle to illustrate the performed analysis. However, this analysis was performed on all of the nine investigated configurations and, at the end of the subsection, the results will be shown for all objects under study.

**Figure 7:**
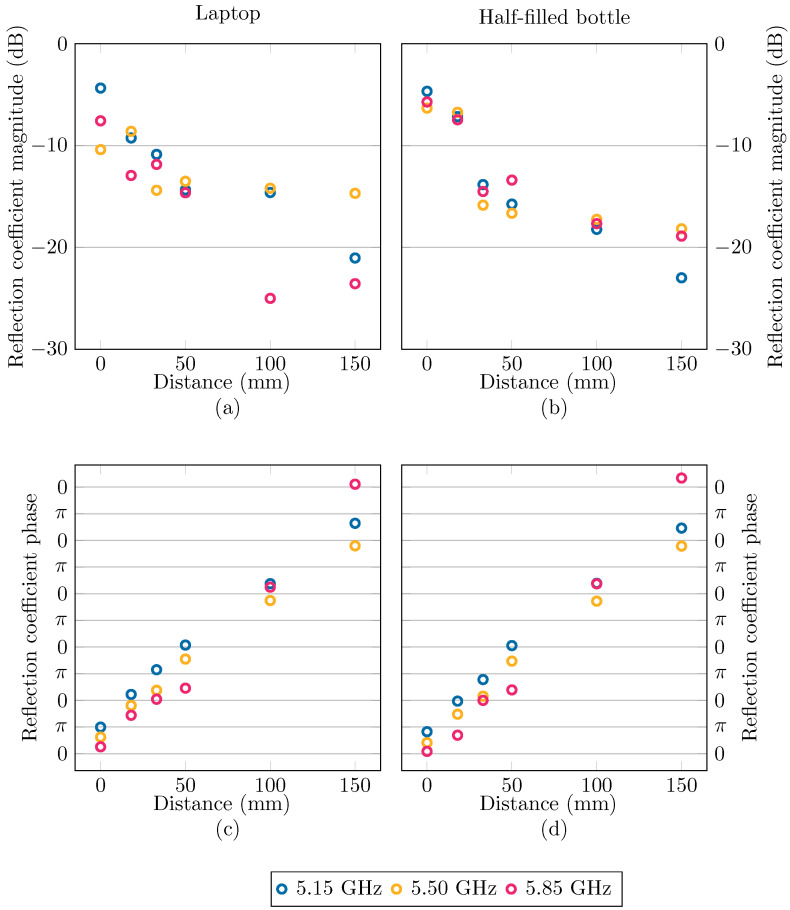
The measured reflection coefficient magnitude, (**a**,**b**), and phase, (**c**,**d**), at the start, the middle, and the end of the $\left[5.15\;\mathrm{GHz},5.85\;\mathrm{GHz}\right]$ band when the laptop, (**a**,**c**), and the half-filled bottle, (**b**,**d**), are placed above the antenna.

As a first step, we split the reflection coefficient Γ into its magnitude |Γ| and phase ∠Γ. Figure 7 shows the reflection coefficient data, split into magnitude and phase, corresponding to the laptop and half-filled bottle. Then, using GPRs, our goal was to make predictions about |Γ| and ∠Γ as a function of frequency *f* and the vertical distance between the object and antenna *d*. In other words, we want to describe the function g(x), where *g* is either |Γ| or ∠Γ, with x = ${\left[f,d\right]}^{T}$.

The main idea behind GPR is the assumption that a vector $g={\left[g\left(x_{1}\right),\ldots ,g\left(x_{N}\right)\right]}^{T}$ containing *N* function values can be described as a sample from an *N*-dimensional normal distribution:

(3) $$g∼N\left(g|0,K\right).$$

The mean of this distribution is commonly set to 0 by normalizing the function *g*. The covariance matrix $K_{ij}=k\left(x_{i},x_{j}\right)$ is defined by the covariance function, or kernel, *k*. The kernel will a priori define the behavior of the function values described by Equation (3), and choosing an appropriate kernel is an important step in the GPR workflow. In this application, we required two different kernels, one for ∠Γ and one for |Γ|. The following kernel was put forward to model the phase ∠Γ:

(4) $$k_{\mathrm{phase}}\left(\left[\begin{array}{c} f_{i}\\ d_{i} \end{array}\right],\left[\begin{array}{c} f_{j}\\ d_{j} \end{array}\right]\right)=\sigma_{g}^{2}\exp\left(-\frac{{\left(f_{i}-f_{j}\right)}^{2}}{2\sigma_{f}^{2}}-\frac{{\left(d_{i}-d_{j}\right)}^{2}}{2\sigma_{d}^{2}}\right).$$

It is an (anisotropic) radial basis function (RBF) kernel, which dictates that two values of the phase will be similar if the frequency values, $f_{i}$ and $f_{j}$, and distance values, $d_{i}$ and $d_{j}$, are also close together [38]. Its hyperparameters are $\sigma_{g}$, which reflect the scale of phase variations, and $\sigma_{f}$ and $\sigma_{d}$, which indicate the length-scales of frequency and distance for which similar phase values can be expected.

For the characterization of |Γ|, a similar kernel is used, albeit with the small difference of an added logarithmic function:

(5) $$k_{\mathrm{mag}}\left(\left[\begin{array}{c} f_{i}\\ d_{i} \end{array}\right],\left[\begin{array}{c} f_{j}\\ d_{j} \end{array}\right]\right)=\sigma_{g}^{2}\exp\left(-\frac{{\left(f_{i}-f_{j}\right)}^{2}}{2\sigma_{f}^{2}}-\frac{{\left(\ln\left(\frac{d_{0}+d_{i}}{1\;\mathrm{mm}}\right)-\ln\left(\frac{d_{0}+d_{j}}{1\;\mathrm{mm}}\right)\right)}^{2}}{2\sigma_{d}^{2}}\right).$$

If the antenna was perfectly matched over the entire frequency band, its reflection coefficient |Γ| would converge to zero as the distance increased and the influence of the objects became less significant. However, when looking at the data in Figure 4a, it is evident that the antenna radiating in free space still has a small, albeit non-zero, reflection coefficient. The magnitude of the reflection coefficient, expressed in dB, does, therefore, not approach −∞ as distance increases but converges to a fixed value. To better describe this behavior, a logarithmic function is introduced in the kernel. Owing to the shape of the logarithmic function, which has a diminishing derivative as its argument increases, the covariance for two different points, $d_{i}$ and $d_{j}$, will be greater when $d_{i}$ and $d_{j}$ are large compared to when they are small. The addition of the parameter $d_{0}$ is intended to shift away from the asymptote of the logarithmic function when the argument approaches zero. This results in the functions described by this kernel having a converging nature at large distances, with the most variation occurring at small distances.

To determine the values of the kernel hyperparameters, given by the vector θ = $\left[\sigma_{g},\sigma_{f},\sigma_{d}\right]$, we tried to find the optimal hyperparameter values that best describe the data. However, the data of ∠Γ and |Γ| include random noise due to instrument limitations, environmental conditions, etc., and it is important to include this noise in our analysis. In general, we assume that the measured values y are normally distributed around the true function values g with a noise variance $\sigma_{n}^{2}$:

(6) $$y∼N\left(y|g,\sigma_{n}^{2}I\right).$$

We can find the optimal values of θ and $\sigma_{n}$ by maximizing the marginal likelihood [37], given by

(7) $$p\left(y|X,\theta ,\sigma_{n}\right)=\int N\left(y|g,\sigma_{n}^{2}I\right)N\left(g|0,K(\theta )\right)dg,$$

for θ and $\sigma_{n}$. All data sets were used during the optimization, so one set of hyperparameters was used for all scenarios. Due to the presence of multiple local maxima in the marginal likelihood for $k_{\mathrm{mag}}$, the value of $d_{0}$ was fixed manually to ensure convergence to hyperparameter values that conformed to the expected behavior. The final hyperparameter values are shown in Table 1.

**Figure 8:**
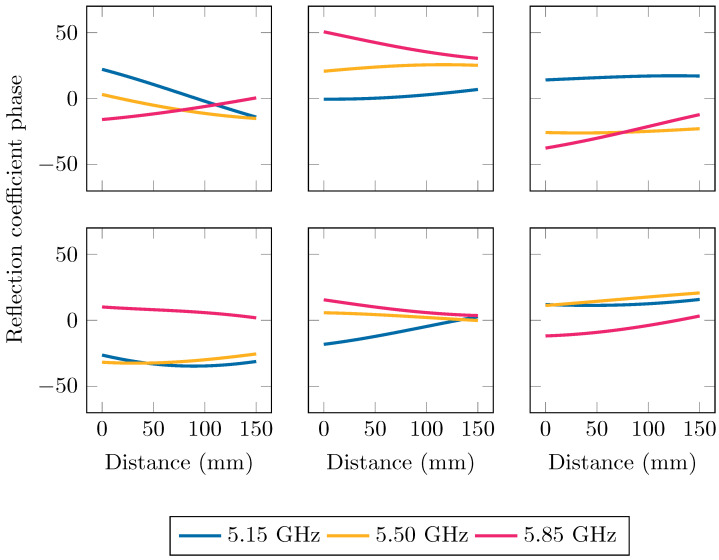
Six functions that were sampled from the distribution in Equation (3), with the $k_{\mathrm{phase}}$ kernel and the hyperparameter values of Table 1, namely $\sigma_{g}=23.5$, $\sigma_{f}=0.243\;\mathrm{GHz}$ and $\sigma_{d}=232\;\mathrm{mm}$. Each sample is a function of both frequency and distance, which is represented with distance-dependent curves at three different frequencies: 5.15 GHz, 5.50 GHz, and 5.85 GHz.

**Figure 9:**
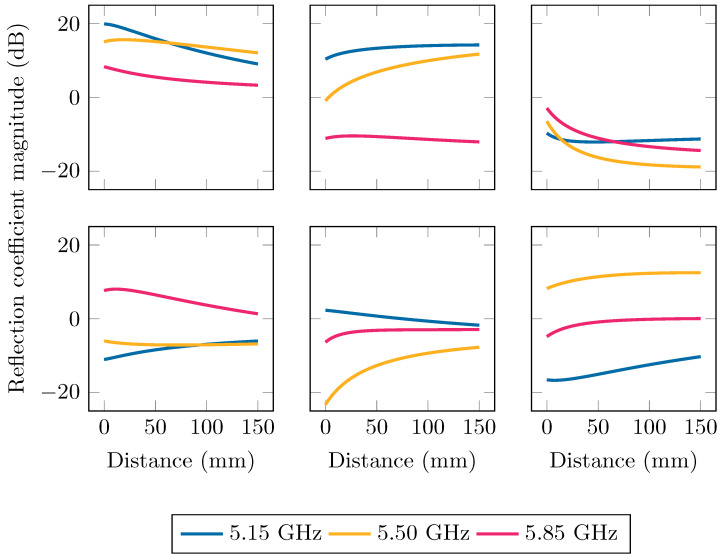
Six functions that were sampled from the distribution in Equation (3), with the $k_{\mathrm{mag}}$ kernel and the hyperparameter values of Table 1, namely $d_{0}=20\;\mathrm{mm}$, $\sigma_{g}=11.1\;\mathrm{dB}$, $\sigma_{f}=0.217\;\mathrm{GHz}$, and $\sigma_{d}=2.54\;\mathrm{mm}$. Each sample is a function of both frequency and distance, which is represented with distance-dependent curves at three different frequencies: 5.15 GHz, 5.50 GHz, and 5.85 GHz.

To illustrate the function behavior that is associated with these kernels in Equations (4) and (5) and the hyperparameter values in Table 1, we can simulate random function realizations that abide to this kernel. This was performed by sampling the distribution of function values given in Equation (3). Six of such functions are shown in Figure 8 and Figure 9 for the phase ∠Γ and magnitude |Γ| of the reflection coefficient, respectively.

As the next step in the GPR algorithm, we expand the distribution in Equation (3) to a joint distribution that includes both the data y that were measured at $X=\left[x_{1},\ldots ,x_{M}\right]$ and the function values $g^{\prime }$ that we want to predict at $X^{\prime }=\left[x_{1}^{\prime },\ldots ,x_{O}^{\prime }\right]$:

(8) $$\left[\begin{array}{c} y\\ g^{\prime } \end{array}\right]∼N\left(\left[\begin{array}{c} y\\ g^{\prime } \end{array}\right]|0,\left[\begin{array}{cc} K+\sigma_{n}^{2}I & K^{\prime }\\ K^{*T} & K^{**} \end{array}\right]\right).$$

In the above equation, the kernel matrix is split up into

$$\begin{array}{cc} \; & K_{ij}=k\left(x_{i},x_{j}\right)\;\mathrm{with}\;x_{i},x_{j}\in X,\\ \; & K_{ij}^{\prime }=k\left(x_{i},x_{j}\right)\;\mathrm{with}\;x_{i}\in X\;\mathrm{and}\;x_{j}^{\prime }\in X^{\prime },\\ \; & K_{ij}^{\prime \prime }=k\left(x_{i},x_{j}\right)\;\mathrm{with}\;x_{i}^{\prime },x_{j}^{\prime }\in X^{\prime }. \end{array}$$

Finally, we transform the joint distribution of y and $g^{\prime }$ into a conditional distribution of $g^{\prime }$ as a function of y [37]:

(9) $$g^{\prime }∼N\left(g^{\prime }|{\bar{g}}^{\prime },\mathrm{cov}\left(g^{\prime }\right)\right),$$

with

(10) $${\bar{g}}^{\prime }=K^{\prime T}{\left[K+\sigma_{n}^{2}I\right]}^{-1}y,$$

and

(11) $$\mathrm{cov}\left(g^{\prime }\right)=K^{\prime \prime }-K^{\prime T}{\left[K+\sigma_{n}^{2}I\right]}^{-1}K^{\prime }.$$

**Figure 10:**
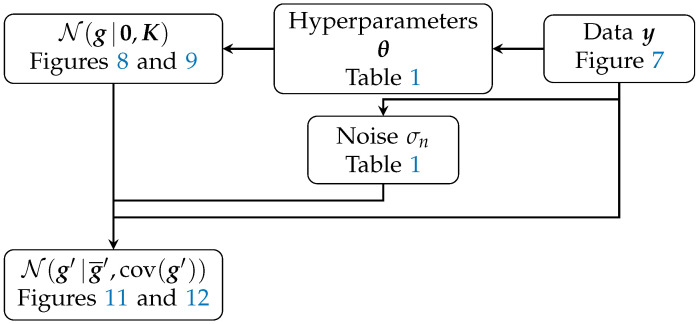
A schematic overview of the different elements in the GPR workflow as it was applied in this research, with references to the Figure 8, Figure 9, Figure 11 and Figure 12 and Table 1 in which the data and results are shown.

In essence, Equation (9) enabled us to predict new function values $g^{\prime }$ that abide by the given kernel *k* and the data y. A schematic summary of the applied GPR algorithm is shown in Figure 10.

**Figure 11:**
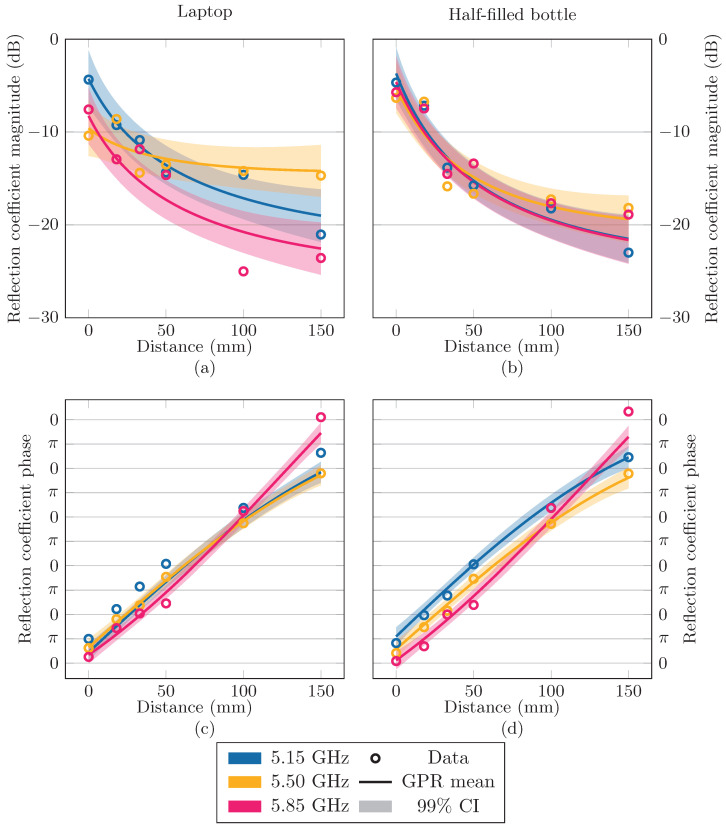
Measured data and the fitted model (with 99% confidence interval) of the phase and magnitude of the reflection coefficient Γ at three different frequencies and a range of distances when a laptop, (**a**,**c**), or a bottle, (**b**,**d**), are placed above the antenna.

**Figure 12:**
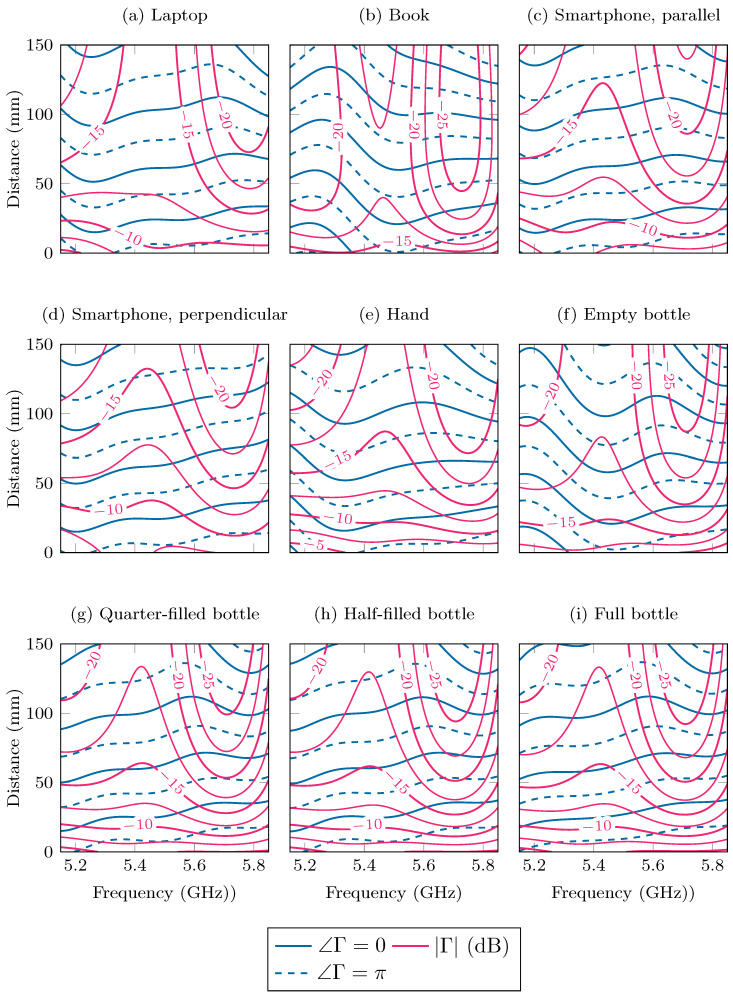
The magnitude and phase of the reflection coefficient Γ of the detuned antenna as a function of the frequency and distance between the object and the antenna, as predicted by the GPR model.

In general, the mean ${\bar{g}}^{\prime }$, as given by Equation (10), is considered to be the best estimator for the true function of interest [37] and is used as the fitted model in the remainder of this paper. As a measure of uncertainty on the function values, a confidence interval (CI) is often given around the mean function, defined by the covariance in Equation (11). In our application, this process was performed twice, once for g=∠Γ with the kernel $k_{\mathrm{phase}}$ and the phase data, and once for g=|Γ| with the kernel $k_{\mathrm{mag}}$ and the magnitude data. For the laptop and half-filled bottle scenarios, the mean functions are shown at the beginning, center, and end frequencies of the band under study in Figure 11, together with the data. In Figure 12, the mean functions are shown for each of the nine scenarios as a function of frequency and distance in a contour plot.

From this analysis, we conclude that the behavior of the reflection coefficient will depend mainly on the type of object that is placed above the antenna. Some objects, such as the book, Figure 12b, and the empty bottle, Figure 12f, will have a relatively small effect, with a reflection coefficient magnitude that is already very small, around −10 to −15 dB, at short distances and that becomes even lower as the distance increases. For other objects, such as the hand, Figure 12e, and the bottles with water, Figure 12g–i, the magnitude of the reflection coefficient starts out high, at −5 dB, but quickly decreases to small values. Furthermore, finally, strongly reflective objects such as the laptop, Figure 12a, and the smartphone, Figure 12c,d, have a reflection coefficient magnitude of around −7.5 dB that decreases relatively slowly as the distance increases, presumably due to significant reflections by these objects.

The effects of the alignment of the smartphone along the slot of the antenna is noticeable within these results. Specifically, the reflection coefficient will decay quicker with increasing distance if the smartphone is placed parallel, Figure 12c, to the antenna slot, in comparison to when it is perpendicular, Figure 12d. This result can be explained by the radiation pattern of the antenna, shown in Figure 4b,c, which is not anisotropic. The antenna radiates more narrowly in the XZ-plane, parallel to the slot. Therefore, if the smartphone is deployed in the radiative near field and far field, and parallel to the slot, it will reflect less of the radiated fields, resulting in a smaller reflection coefficient.

Adding water to the bottle has a significant effect on the behavior of the reflection coefficient. In particular, when the bottle contains water, Figure 12g–i, the reflection coefficient will have a larger magnitude compared to the empty bottle, Figure 12f. The amount of water does seem to have little effect, as the graphs in Figure 12g–i are very similar.

### 3.2. Reliability Optimization

To simulate a dynamic environment, random position shifts in the reflecting objects were introduced. The variations in position were translated into (frequency-dependent) variations in the reflection coefficient Γ by evaluating the GPR-fitted models, defined by Equation (10), for ∠Γ and |Γ| and for each *d* and *f* value. These variations in Γ were, in turn, translated into variations in $S_{11}$ and $G_{T}$, through Equations (1) and (2).

The system reliability *R*, defined by

(12) $$R\left(C\right)=\int_{0}^{\infty }h\left(C,d\right)p\left(d\right)dd\approx \frac{1}{N}\sum_{j=1}^{N}h\left(C,d_{j}\right)$$

with

(13) $$h\left(C,d\right)=\left\{\begin{array}{cc} 1 & G_{T}>G_{T,\min}\;\mathrm{and}\;|S_{11}\left|<\right|S_{11}{|}_{\max}\;\;\forall f_{k}\\ 0 & \mathrm{otherwise}, \end{array}\right.$$

is the probability that $|S_{11}|$ and $G_{T}$ abide to a certain upper and lower bound, within a certain frequency band. In practice, this is calculated by Monte Carlo integration using Latin hypercube samples $d_{j}$ from the probability density of the distance p(d) [39]. The frequency band is reduced to a set of equidistant discrete frequencies $f_{k}$.

The goal is to obtain the optimal capacitance values C = $\left[C_{1},C_{2},C_{3}\right]$ of the three varactors that maximize R(C). The optimal value of C will depend on the environmental factors, such as the distance distribution p(d) and the type of interfering object (bottle or laptop), but also on the system prerequisites, such as the required frequency band, $G_{T,\min}$ and $|S_{11}{|}_{\max}$. The optimization was accomplished using a combination of the Nelder–Mead method and a basin-hopping technique. The Nelder–Mead method is a direct search method that makes no use of the derivatives of the cost function [40], which is crucial since R(C) has no closed form and thus its derivatives are not known. The Nelder–Mead algorithm is only able to find local maxima and, in the case of the presence of multiple local maxima, it will converge differently depending on the starting position of the search. During our research, R(C) proved to have multiple local maxima and thus basin-hopping was employed to perform multiple local optimizations, each with a different starting position, in order to increase the probability of finding the global maximum [41].

During the simulations, the frequency band was divided into five equidistant frequencies. The value five was chosen to limit computation time and because further discretization did not lead to better results. The number of samples *N* was set to 100 during the search for local maxima and to 1000 at the end, to fine-tune the global maximum. This optimization strategy will be referred to as Opt-R.

## 4. Application

The distance distribution p(d) is dependent on the specific conditions in which the antenna and matching network are deployed. As an illustrative example and a proof of principle, we designate a (rescaled) beta distribution to the displacement of the scattering object:

(14) $$p\left(d|a,b\right)=\frac{1}{B(a,b)}{\left(\frac{d}{d_{\max}}\right)}^{a-1}{\left(1-\frac{d}{d_{\max}}\right)}^{b-1},$$

where the beta function B is the normalizing constant of the distribution and $d_{\max}$ is the maximum distance equal to 150 mm.

**Figure 13:**
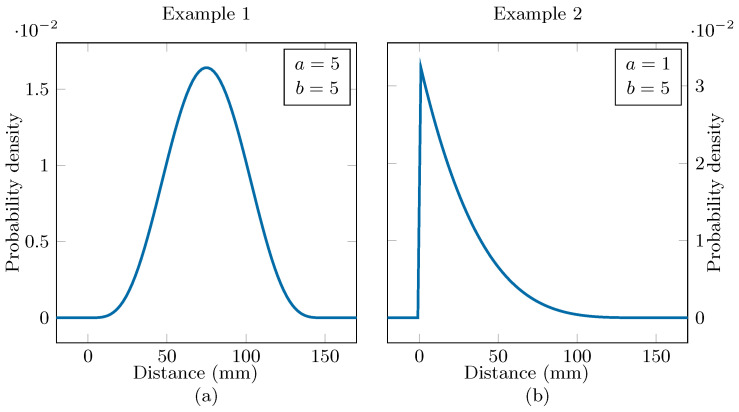
Beta distributions used in Example 1 (**a**) and Example 2 (**b**).

Two examples are considered: in Example 1, the distribution parameters are a=5 and b=5, which results in a symmetrical distribution with a mean at 75 mm, where the presence of the objects is not that influential. As a counterexample, the parameters in Example 2 are a=1 and b=5. This results in a non-symmetrical distribution with a mode at d=0mm, where the reflection coefficient is larger and varies more. The beta distributions for both examples are shown in Figure 13. In Example 1, the operational band is the full $\left[5.15\;\mathrm{GHz},5.85\;\mathrm{GHz}\right]$ band. Example 2 poses a more challenging matching scenario and, therefore, the operational band is reduced to the $\left[5.5\;\mathrm{GHz},5.65\;\mathrm{GHz}\right]$, IEEE 802.11–114 channel. For both examples, two different sets of prerequisites are considered, one where $G_{t}>-2\;\mathrm{dB}$ and $|S_{11}|\;<-15\;\mathrm{dB}$ is desired, and one more lenient set with $G_{t}>-3\;\mathrm{dB}$ and $|S_{11}|\;<-10\;\mathrm{dB}$.

As mentioned earlier, it is not always possible or preferable to have an implementation that follows the changes in the reflection coefficient as they occur. Therefore, as a comparison with the novel strategy introduced in Section 3.2, a typical (deterministic) optimization strategy was applied where random variations were not considered. This strategy was to maximize the transducer gain $G_{t}$ with the prerequisite that $|S_{11}|$ was matched throughout the band and the reflection coefficient was only considered at the average distance. This strategy is referred to as Opt-G.

## 5. Results

The results of the simulations are presented in Table 2. First, the impact of the dynamic environment on the system reliability using the classic Opt-G algorithm is discussed and, afterwards, the improvements in reliability that Opt-R offers are analyzed.

### 5.1. Impact of the Dynamic Environment

According to Table 2, not taking into consideration the variations in impedance mismatch due to a dynamic environment, as with Opt-G, can have a dramatic effect on the reliability and performance of the matching network, even when variations of only a few centimeters are considered. This decrease in reliability is the largest in Example 2, even though a smaller band is considered in this example. Looking at Figure 12, we can see that the biggest variation in the reflection coefficient amplitude is below 50 mm, which is where most of the distance variation is located in Example 2.

The type of object does also have an impact on the system reliability. In Example 1, where the distance distribution is given by Figure 13a, the biggest factor that affects the reliability is the value of the reflection coefficient at values around 75 mm, where the mean of the distance distribution lies. Therefore, the laptop and the smartphone perform the worst, as they have the largest reflection coefficient at these distances, followed by the hand and the bottles containing water and the book and empty bottle, which have only a small effect on the reliability. In Example 2, the distribution of the object distance is given by Figure 13b. In this example, the behavior of the reflection coefficient at short distances, below 50 mm, is important and objects that are associated with a high reflection coefficient when placed very close to the antenna yield bad reliability, even when the reflection coefficient decays very quickly as the distance increases. Examples of such objects are the hand and the bottles containing water.

As is to be expected from the results of Section 3.1, the smartphone had the largest impact on reliability when it was placed perpendicular to the slot. Additionally, filling the bottle with water did also decrease the reliability.

### 5.2. Improvements to Proposed Strategy

**Figure 14:**
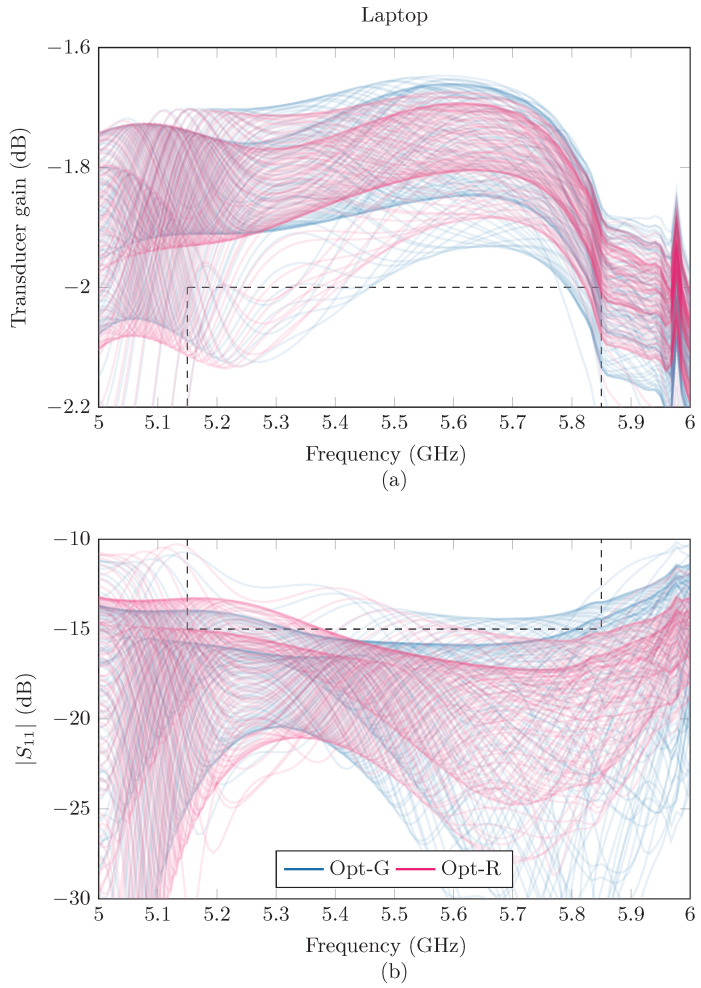
$G_{t}$ (**a**) and $|S_{11}|$ (**b**) curves resulting from the Opt-G (blue) and Opt-R (purple) strategy in Example 1, with $G_{t}>-2\;\mathrm{dB}$ and $|S_{11}|\;<-15\;\mathrm{dB}$ and with the laptop placed above the antenna. The prerequisites are indicated with dashed lines.

**Figure 15:**
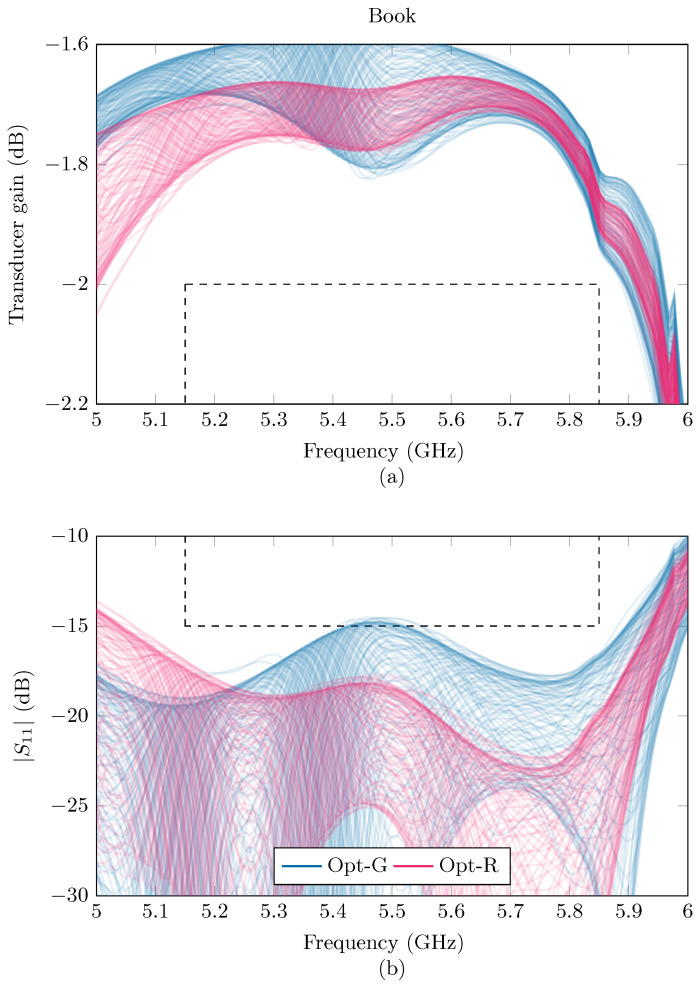
$G_{t}$ (**a**) and $|S_{11}|$ (**b**) curves resulting from the Opt-G (blue) and Opt-R (purple) strategy in Example 1, with $G_{t}>-2\;\mathrm{dB}$ and $|S_{11}|\;<-15\;\mathrm{dB}$ and with the book placed above the antenna. The prerequisites are indicated with dashed lines.

In the considered examples, the proposed strategy, Opt-R, increases the reliability by 10% to 40%, until the ceiling of 100% is reached, as can be seen in Table 2. This gives an indication that taking into account these random deviations can give a boost to the reliability of a matching network when it is implemented in a dynamic environment. A visualization of the effects of the two different optimization strategies in Example 1 are given in Figure 14 for the laptop and Figure 15 for the book. By looking carefully at the different realizations of the $G_{T}$ and $|S_{11}|$ curves, one can see that the Opt-R strategy succeeds in actively trying to minimize the number of curves that cross the predetermined bounds.

## 6. Conclusions

From the performed characterization of the reflection coefficient at the port of an antenna element that was detuned by disturbing objects, we could conclude that the reflection coefficient displays a large frequency dependency. This decreases the odds of reliably matching an entire band by only considering its center frequency. Matching on an entire frequency band at once is computationally more expensive and, combined with the possibility for accidental modulation, real-time matching that adapts to the dynamic changes in the reflection coefficient due to environmental factors becomes challenging.

By means of simulation, we were able to show that non-real-time deterministic matching, which does not react to a variable environment, will suffer from a severe reduction in reliability. This reduction in reliability is dependent on the type of object, but also on its orientation with respect to the antenna. We introduced a stochastic matching strategy that prioritizes robustness when confronted with a variable reflection coefficient and, using this novel strategy, were able to increase the reliability by 10% to 40%.

The proposed strategy was defined in a broad way and can in principle be applied to any matching network that suffers from the effects of a variable and unreliable environment. We expect that, in the future, stochastic matching strategies can be used to make the integration of antennas in smart surfaces more viable. The optimization strategy could also be extended to be applicable to a broader range of situations at once, such as multiple different objects with different orientations. This could make the matching even more robust and could allow for spreading out the matching routines further in time. Additionally, further research could investigate how using multiple antennas in smart surfaces for IoT applications could increase the system reliability in the form of an antenna array or an antenna diversity scheme.

## Figures and Tables

**Table 1:** The optimized hyperparameters of kernels $K_{\mathrm{phase}}$ and $K_{\mathrm{mag}}$. The optimization was performed by maximizing the marginal likelihood, given in Equation (7), except for $d_{0}$, which was fixed manually.

	$d_{0}$	$\sigma_{g}$	$\sigma_{f}$	$\sigma_{d}$	$\sigma_{n}$
$K_{\mathrm{phase}}$	n.a.	23.5	0.243 GHz	232 mm	1.07
$K_{\mathrm{mag}}$	20 mm	11.1 dB	0.217 GHz	2.54	2.61 dB

**Table 2:** The reliability of the tuned matching network, using the two optimization strategies in both examples, with multiple types of disturbing objects. Two different sets of prerequisites are considered. The percentages in bold text correspond to the settings shown in Figure 14 and Figure 15.

		$G_{T}>-2$ $|S_{11}|\;<-15$		$G_{T}>-3$ $|S_{11}|\;<-10$	
		**Opt-G**	**Opt-R**	**Opt-G**	**Opt-R**
Example 1 a=5, b=5 5.15 to 5.85 GHz	Laptop	**43.7%**	**54.1%**	100%	100%
	Book	**85.4%**	**100%**	100%	100%
	Smartphone, parallel	46.3%	54.8%	100%	100%
	Smartphone, perpendicular	17.7%	33.0%	99.5%	99.9%
	Hand	66.9%	81.5%	99.9%	99.9%
	Empty bottle	91.5%	99.4%	100%	99.9%
	Quarter-filled bottle	74.3%	79.3%	100%	99.9%
	Half-filled bottle	72.9%	79.1%	100%	99.9%
	Full	70%	79.5%	100%	100%
Example 2 a=1, b=5 5.50 to 5.65 GHz	Laptop	27.2%	50.5%	79.4%	100%
	Book	56.8%	93.3%	100%	100%
	Smartphone, parallel	1.7%	5.1%	69.5%	80.3%
	Smartphone, perpendicular	0.0%	1.7%	51.1%	66.5%
	Hand	0.0%	11.3%	47.6%	71.5%
	Empty bottle	16.0%	43.0%	100%	100%
	Quarter-filled bottle	10.8%	11.6%	67.4%	71.3%
	Half-filled bottle	3.2%	9.8%	66.0%	70.5%
	Full bottle	0.5%	13.1%	71.8%	75.8%

## Data Availability

The original contributions presented in the study are included in the article, further inquiries can be directed to the corresponding author.
